# Detecting introgressive hybridization to maintain genetic integrity in endangered large waterbird: a case study in milky stork

**DOI:** 10.1038/s41598-023-35566-x

**Published:** 2023-06-01

**Authors:** Damisa Kaminsin, Natapot Warrit, Rangsinee Sankhom, Krittee Piamsanga, Saowaphang Sanannu, Sudarath Baicharoen, Amporn Wiwegweaw

**Affiliations:** 1 Population and Conservation Genetics Laboratory, Department of Biology, Faculty of Science, Chulalongkorn University, Bangkok, 10330 Thailand; 2 Center of Excellence in Entomology and Department of Biology, Faculty of Science, Chulalongkorn University, Bangkok, 10330 Thailand; 3 Durrell Institute of Conservation and Ecology, School of Anthropology and Conservation, University of Kent, Canterbury, Kent, CT2 7NR UK; 4 Genomic Resource Center, Animal Conservation and Research Institute, The Zoological Park Organization of Thailand, Bangkok, 10300 Thailand; 5 Genetic Center, Animal Conservation and Research Institute, The Zoological Park Organization of Thailand, Bangkok, 10300 Thailand

**Keywords:** Evolution, Genetics, Molecular biology

## Abstract

Hybridization between milky stork (*Mycteria cinerea*) and painted stork (*M. leucocephala*) occurs frequently in captivity. Dusit Zoo is a captive breeding facility where storks with phenotypically ambiguous patterns have recently been observed, and their status remaining inconclusive. Here, we used a combination of phenotypic characters and genetic markers (cytochrome *b* and 14 microsatellite markers) to distinguish and identify hybrids from the two parental species (*n* = 114). Haplotype analysis revealed asymmetric mtDNA introgression from *M. cinerea* to *M. leucocephala*, with twelve morphologically classified *M. leucocephala* individuals carrying heterospecific mtDNA. Comprehensive biparental genetic assessments identified 33% of all three genetic clusters as admixed individuals, of which most were either F2 hybrids, backcrosses with *M. leucocephala*, or hybrids of unknown generation, implying weak premating isolation with the absence of intrinsic postzygotic isolation between parentals. Morphological analysis demonstrated that the absence or indistinctness of a black bar across the breast is the most noticeable trait to identify these hybrids. The endangered *M. cinerea* was found to have genomic contamination from *M. leucocephala* and vice versa, with at least 41 hybrid individuals being identified. These findings provide critical information for detecting hybrids and identifying suitable breeding stocks with genetic purity for future reintroduction and conservation management.

## Introduction

Hybridization and its impact on the evolutionary consequences for hybridizing species are heavily reliant on the fate of their hybrid offspring, because fertile hybrids can mediate gene flow or introgression of genetic materials between species^1,2^, although this may not indicate the flow of functional or ecologically meaningful genes. This process is regarded as one of the major threats to species that are highly threatened or have a small population, as it can lead to the decline or eventual extinction of the pure species’ genetic background (genetic swamping). Accordingly, the emergence of hybrids tends to be an issue because they appear to have detrimental consequences for pure species^3–6^.

Ex situ management, in terms of captive breeding, has become a conservation strategy for the rescue and restoration of an endangered species in preparation for future reintroduction^7–9^. However, unintentional human-mediated hybridization may detrimentally influence the success of breeding and reintroduction, raising serious concerns about the genetic integrity of breeding stocks or reintroduced individuals^2,10–12^. Detecting introgressive hybridization is, therefore, an urgent need for preserving the genetic integrity of the remnant pure species.

The milky stork (*Mycteria cinerea*) is a large waterbird in the Ciconiidae family with an almost entirely white plumage that is patchily distributed in tropical wetland ecosystems across Southeast Asia (Cambodia, Indonesia, and Malaysia)^13–15^. The expansion of habitat degradation and environmental pollution, primarily caused by anthropogenic activities, has dramatically decreased *M. cinerea* populations, leading them to be listed as an endangered species on the IUCN Red List, with approximately 1500 mature individuals remaining in the wild^16,17^. In Thailand, *M. cinerea* was once found in the south, but was thought to be locally extinct in the past few decades^18^. Thus, most of the storks found nowadays in Thailand are vagrants, typically found in the central region.

The Zoological Park Organization of Thailand has initiated a project to breed *M. cinerea* in captivity prior to future reintroduction in Thailand^19,20^*.* The primarily captive breeding of *M. cinerea* took place in 1998 at Dusit Zoo (DZ), Bangkok, where 19 founders were raised together with its sister taxon, the near threatened painted stork (*M. leucocephala*), for which captive breeding had already been commenced in 1997 with an initial 70 founding individuals. The two stork species shared a large aviary for 16 years until all captive bred *M. cinerea* (*n* = 21) were relocated to Nakhon Ratchasima Zoo (NRZ: Nakhon Ratchasima Province) in 2014 for further breeding while *M. leucocephala* were still maintained in the DZ.

The two storks exhibit similarities in their appearance, behavior, and have a close evolutionary relationship with little genetic divergence between them^21–23^. Morphologically, *M. leucocephala* can be distinguished from *M. cinerea* by plumage patterns and colorations, including black and white markings on the wings, a black bar across the breast, and a deep pinkish on the tertials^22,23^. These three distinct traits are present only in *M. leucocephala* but not in *M. cinerea*. To date, the captive breeding program at DZ has increased the number of *M. leucocephala* to 173 individuals, while at NRZ the *M. cinerea* population has been expanded to 48 individuals.

It has recently been observed that some stork individuals at DZ showed phenotypically ambiguous patterns and colors (intermediate traits between *M. cinerea* and *M. leucocephala*) and could not be clearly identified through their morphology. Therefore, these stork individuals were suspected to be hybrids of the two species, as previously reported in the Malaysian National Zoo (Malaysia) and Jurong Bird Park (Singapore)^24,25^. Here, we combined phenotypic characteristics and genetic analyses (both maternally and biparentally inherited DNA) to identify and discriminate captive hybrids from *M. cinerea* and *M. leucocephala*. The findings will provide fundamental information for developing conservation management and reintroducing the stork population.

## Results

### Morphological analysis

**Figure 1:**
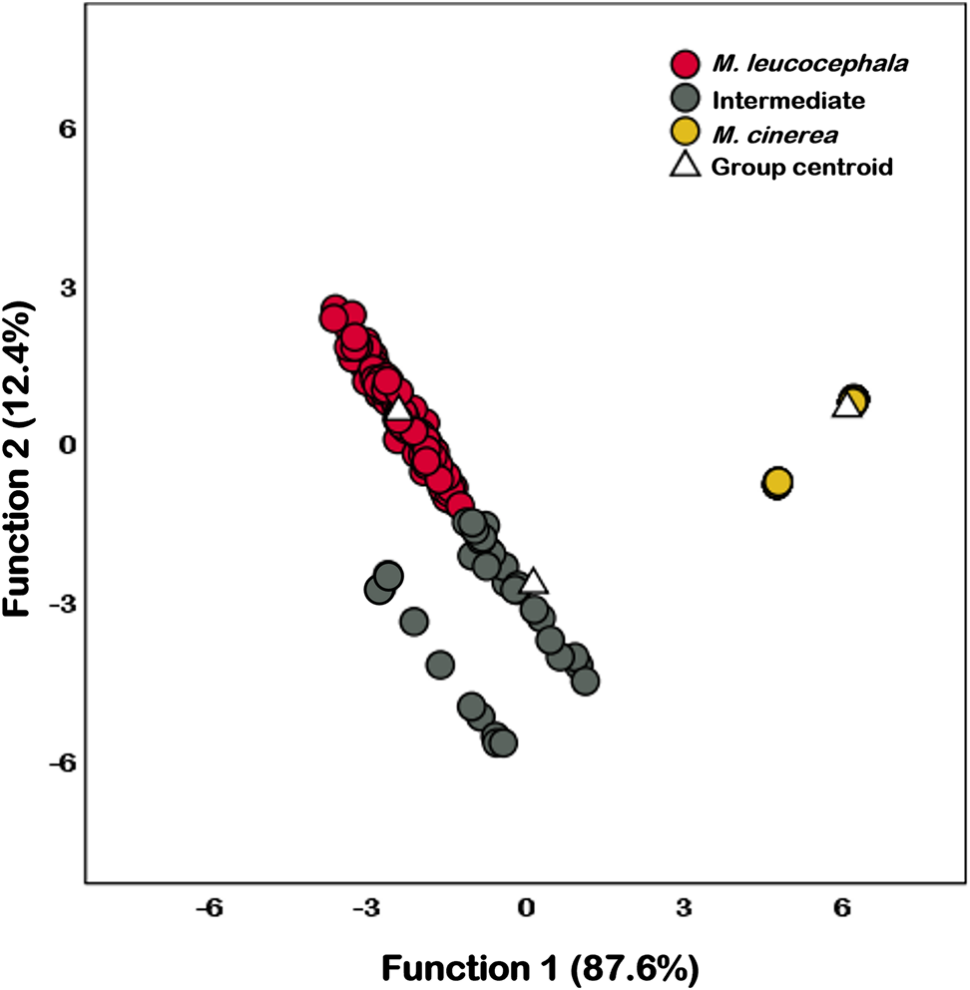
Canonical discriminant function plot of discriminant analysis of phenotypically classified *M. leucocephala* (red dot), intermediate individuals (grey dot), and *M. cinerea* (yellow dot) based on six phenotypic variables. The dots were colored following the predicted groups from the analysis.

The canonical discriminant function (CDF) plot generated by discriminant analysis clearly showed three distinct groups consisting of phenotypically *M. leucocephala*, *M. cinerea*, and intermediate groups (Fig. 1). The corresponding percent of variance for CDFs 1 and 2 were 87.6% and 12.4%, respectively, with CDF 1 representing black and white markings on the wings (MOW), pink tertial feathers (PTF), all-white body (AWB), and black and white markings under the wings (MUW), while CDF 2 represents a black bar across the breast (BBB) and pink/coral markings under the wings (PMUW). The eigenvalues and significant characters used in constructing the plot are provided in Supplementary Table S1.

The plot revealed a separation between the *M. leucocephala* and *M. cinerea* groups, with the *M. leucocephala* group predominantly on the negative side of CDF 1 and *M. cinerea* group showing the opposite. In contrast, the intermediate individuals exhibited a wide range of phenotypic traits between the two pure species and were positioned closer to the *M. leucocephala* group than the *M. cinerea* group (Fig. 1). However, nine intermediate individuals (ID: IN17, IN32, IN74, IN123, IN128, IN156, IN163, IN166, and IN168) were separated from the main intermediate group and placed on the negative side of CDF 1. This could be due to the presence of PMUW, which was missing from the main intermediate group. Based on these six phenotypic variables, the birds were tentatively classified as 122 *M. leucocephala* (57.55%), 53 *M. cinerea* (48 individuals from NRZ and five individuals from DZ, 25.00%), and 37 intermediate individuals (17.45%).

### Phylogenetic analyses on mtDNA data

**Figure 2:**
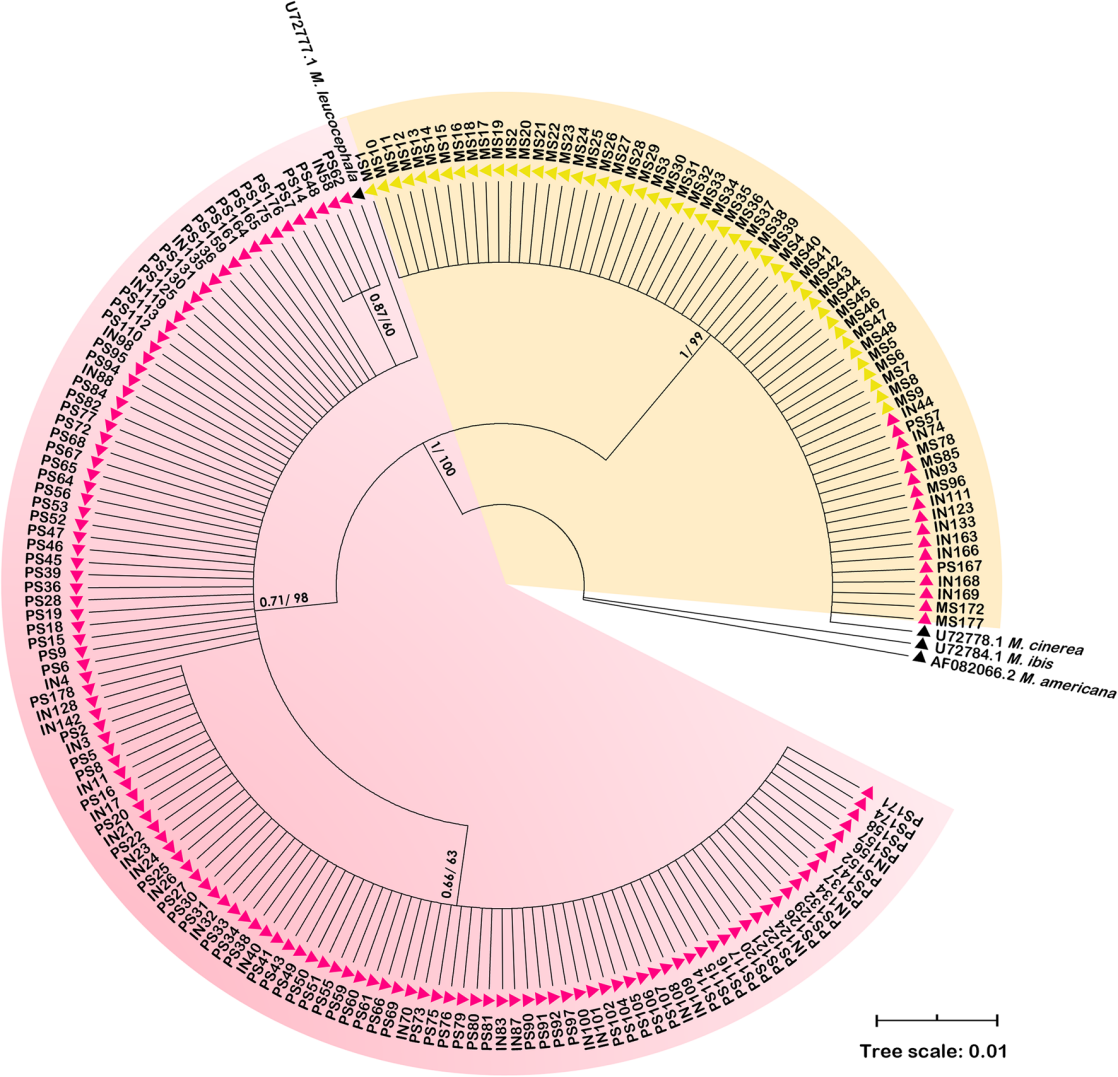
Phylogenetic tree reconstructed from the *cyt b* sequences. The posterior probability and bootstrap values, analyzed by BI and ML methods, are shown at each node. PS, IN, and MS represent morphologically identified *M. leucocephala*, intermediate individuals, and *M. cinerea*, respectively. Clades that contain individuals with mtDNA haplotypes of *M. leucocephala* and *M. cinerea* are highlighted in pink and yellow, respectively. Individuals from Dusit and Nakhon Ratchasima Zoos are represented by pink and yellow triangles. This tree was generated and edited using MEGA v.11.0.6^50^.

A total of 190 individuals, comprised of 100 *M. leucocephala*, 37 intermediate individuals, and 53 *M. cinerea,* from the morphological identification, were sequenced for the mitochondrial cytochrome *b* (*cyt b*) gene, yielding a 1029 bp length sequence with no internal stop codons. Both Bayesian inference (BI) and maximum likelihood (ML) phylogenetic trees revealed similar topologies and clearly demonstrated two major lineages between *M. leucocephala* and *M. cinerea* groups with significant support values (Fig. 2). The intermediate individuals were nested within these two clades. In the *M. cinerea* lineage, the mtDNA sequences showed only one haplotype, whereas the *M. leucocephala* lineage showed three haplotypes that differed by 1–2 bp, with the most frequent haplotype being represented in 78 (62.40%) individuals.

Interestingly, 10 intermediate individuals whose morphology was closer to that of *M. leucocephala* (IN44, IN74, IN93, IN111, IN123, IN133, IN163, IN166, IN168, and IN169) and two morphologically *M. leucocephala* individuals (PS57 and PS167) had the *M. cinerea* haplotype, confirming that these individuals were hybrids of the two stork species (Fig. 2). The other 27 intermediate individuals had *M. leucocephala* haplotypes and so the mtDNA data alone was insufficient to definitively confirm their hybrid identity as they could be either pure *M. leucocephala* or hybrids. Additionally, five storks from DZ (MS78, MS85, MS96, MS172, and MS177) that were morphologically identified as *M. cinerea* carried the *M. cinerea* mtDNA haplotype, implying that they are more likely pure *M. cinerea*.

### Population structure and hybridization analyses on microsatellite data

We genotyped 14 microsatellite loci for each of the 114 individuals (24 *M. leucocephala*, 37 intermediate individuals, and 53 *M. cinerea* according to morphological identification) whose genomes were amplified successfully by all primer pairs in cross-species amplification. However, we found that locus Cc06 was unreliable for allelic reading, and three loci (Cbo133, Cbo235, and Cc05) were monomorphic; thus, these loci were excluded from the analyses (see Supplementary Table S2). There was no evidence of linkage disequilibrium in any of the loci comparisons when Bonferroni correction was used with a significance level of 0.01. Loci Cbo168, Cc10, Cc58, and Cc72 deviated significantly from Hardy–Weinberg equilibrium (HWE) (*P* < 0.05). However, they were not excluded from further analyses because the existence of HWE deviation could be linked to a recent genetic admixture between two populations in DZ, as evidenced by the presence of potential hybrid individuals.

**Figure 3:**
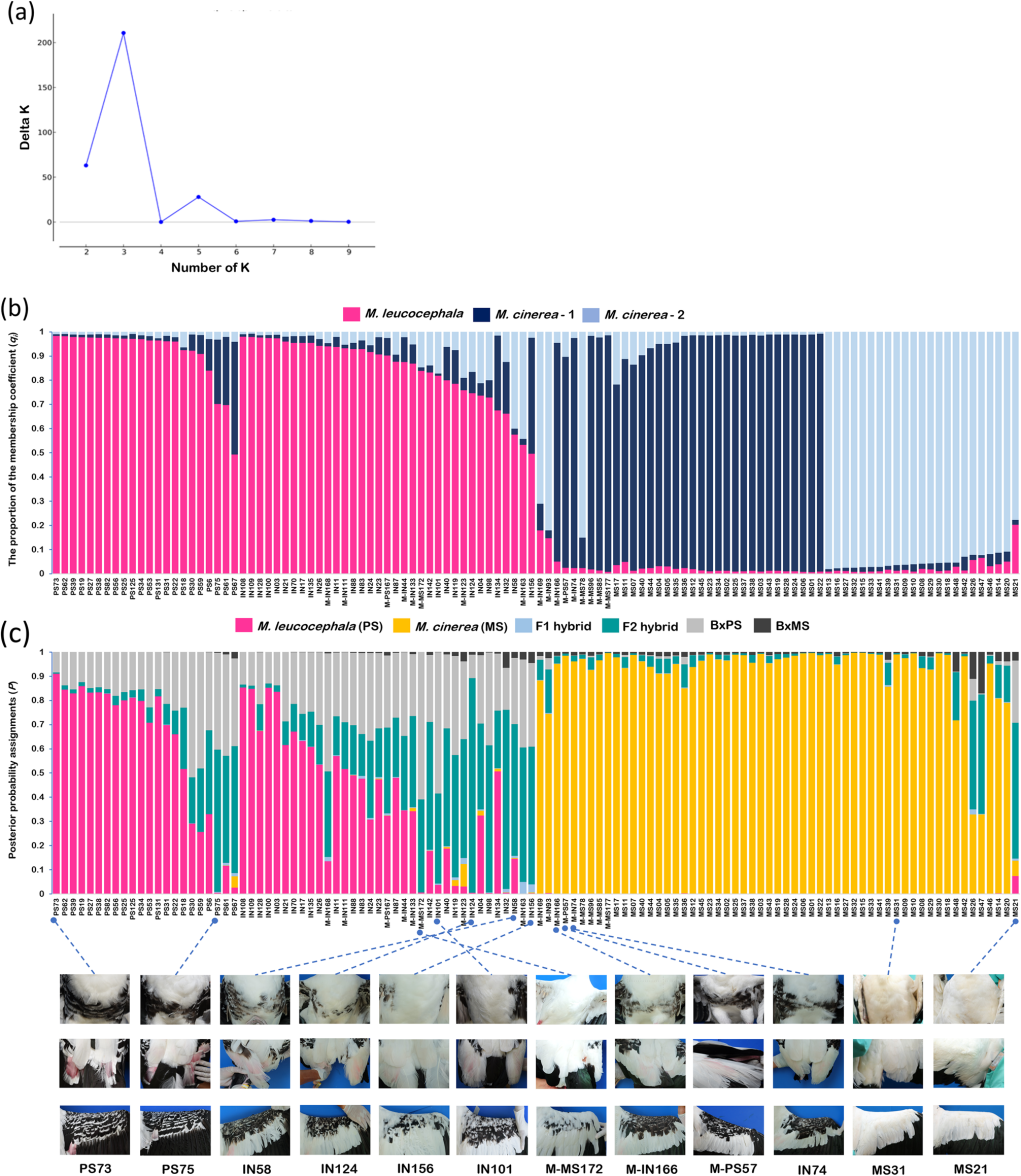
Population structure of 114 individuals (24 *M. leucocephala*, 37 intermediate individuals, and 53 *M. cinerea* based on morphology). (**a**) Evanno’s ΔK graph suggesting K = 3. (**b**) Genetic clusters analyzed by STRUCTURE based on microsatellite markers with inferred K = 3 and inferred clusters are shown in a different color. Each vertical bar represents the proportion of the membership coefficient (*qi*) of each individual. (**c**) Individual genotype classification analyzed by NEWHYBRIDS. Each column represents the posterior probability (*P*) of each individual to belong to six different genotypic classes. PS, IN, and MS represent morphologically identified *M. leucocephala,* intermediate individuals, and *M. cinerea*, respectively. Individuals from DZ that carried the *M. cinerea* mtDNA haplotype are designed by an M.

STRUCTURE analysis of the ten polymorphic microsatellite loci revealed the highest Delta K (ΔK) for K = 3, indicating that the individuals could be divided into three genetic clusters (see Supplementary Table S3 and Fig. 3a). Overall, STRUCTURE analysis detected 38 individuals (eight *M. leucocephala*, 26 intermediate individuals, and four *M. cinerea,* see Supplementary Table S4) that showed signs of having a genetic admixture (Fig. 3b).

NEWHYBRIDS was subsequently used to evaluate the existence of hybrids between the two storks. Of 24 individuals morphologically identified as *M. leucocephala*; 16 individuals were classified as pure *M. leucocephala,* two individuals (PS67 and PS75) were classified as F2 hybrids, one individual (PS30) was classified as a backcross with *M. leucocephala*, one individual (PS57) was classified as *M. cinerea*, and four individuals (PS06, PS59, PS61, and PS167) were classified as hybrids of unknown generation. From 37 individuals morphologically identified as intermediate; 13 individuals were classified as pure *M. leucocephala*, eight individuals were classified as F2 hybrids, four individuals (IN74, IN93, IN166, and IN169) were classified as *M. cinerea*, one individual (IN101) was classified as a backcross with *M. leucocephala*, and 11 individuals were classified as hybrids of unknown generation. From 53 individuals morphologically identified as *M. cinerea*; 49 individuals (45 individuals from NRZ and four from DZ) were classified as pure *M. cinerea*, two individuals (MS26 and MS47) were classified as hybrids of unknown generation, one individual (MS21) was classified as an F2 hybrid, and one individual (MS172 from DZ) was classified as a backcross with *M. leucocephala*. Thus, a total of 114 individuals could be identified according to their genotype classes as 54 pure *M. cinerea* (47.37%), 29 pure *M. leucocephala* (25.44%), 17 hybrids of unknown generation (14.91%), 11 F2 hybrids (9.65%), and three backcrosses with *M. leucocephala* (2.63%), but with no F1 hybrids or backcrosses with *M. cinerea* (Fig. 3c).

Comparing the results from STRUCTURE and NEWHYBRIDS, most results were highly consistent, with the exception of five individuals (IN11, IN26, IN111, IN134, and PS18), which were classified as admixed by STRUCTURE but as pure *M. leucocephala* by NEWHYBRIDS (see Supplementary Table S4). We hypothesized that these individuals were hybrids that had been backcrossing with *M. leucocephala* for multiple generations rather than pure *M. leucocephala* because they do not appear to be pure *M. leucocephala* phenotypically. In some storks, however, there was inconsistency between the morphological and genetic data (IN03, IN17, IN21, IN70, IN100, IN108, IN109, IN128, and IN135). Conversely, four DZ individuals (MS78, MS85, MS96, and MS177) showed congruence between the morphological and genetic data, indicating they were neither pure *M. leucocephala* nor hybrids (see Supplementary Table S4), but rather they were pure *M. cinerea* that had not been relocated to NRZ in 2014. Since the juveniles of the two species appeared strikingly similar, it is likely that they were still juveniles in 2014 and were misidentified as juvenile *M. leucocephala*.

## Discussion

### Introgressive hybridization between *M. cinerea* and *M. leucocephala*

Detecting hybrids from the pure species is a critical first step in developing a breeding program for endangered or extinct in the wild species for subsequent reintroduction in the future^26,27^. This study shows the first evidence of introgressive hybridization between *M. cinerea* and *M. leucocephala* in Thailand, where the two storks were historically kept and raised together in the same captivity in DZ. The DZ was made aware of the problem and is the only zoo actively attempting to prevent hybridization by keeping the two species separate, even if their efforts were not completely successful. The presence of hybrids among pure parental populations as a result of past hybridization raises concerns about the genetic integrity of breeding stock, which needs to be explicitly investigated.

Morphological based CDF analysis highlighted that *M. cinerea* and *M. leucocephala* could be distinguished by six phenotypic characters, whereas intermediate individuals (putative hybrids) were placed in a middle position between the two groups. According to the plot in Fig. 1, *M. leucocephala* and the intermediate groups showed more data dispersion than *M. cinerea*, which could be attributed to the wide variation in the BBB, MOW, and PTF traits. In contrast, *M. cinerea* lacks these three characteristics, they exhibit AWB and an absence or indistinct MUW.

Without explicit examination of hybrid production, genomic introgression may possibly reflect the direction of hybridization success between species and help us understand the evolutionary history and mechanisms of reproductive isolation between hybridizing species^28–30^. In this study, haplotype analysis detected *M. cinerea* haplotypes in several morphologically similar *M. leucocephala* but not vice versa, inferring asymmetric introgression of mtDNA from *M. cinerea* to *M. leucocephala* populations. Thus, the F1 hybrid is most likely produced successfully by *M. cinerea* females. However, we found either species’ mtDNA haplotype in F2 hybrid genomes, raising the possibility of successful reciprocal hybridization across species. Since no first-generation hybrid was identified in this study, the direction of hybridization success between these incompletely isolated species remains uncertain. However, there appears to be no intrinsic postzygotic isolation; F1 hybrids are viable and not sterile, as evidenced by the presence of F2 and backcross hybrids; and the low sequence divergence of the *cyt b* gene supported this^21^.

STRUCTURE analysis revealed three genetic clusters (K = 3) among the examined storks, one in the *M. leucocephala* population and two in the *M. cinerea* population. Although we do not have enough information to draw conclusions about their origins, it is probable that the founders of *M. cinerea* came from two distinct sources. STRUCTURE analysis with various threshold values was performed. A threshold of < 0.95 (i.e., a *qi* of 0.8) lead to an underestimation of hybrids due to mtDNA data inconsistency. In contrast, with a *qi* of 0.99^25^, all birds were classified as admixed (an overestimation of admixed individuals), because the highest *qi* values of pure *M. cinerea* and *M. leucocephala* were 0.983 and 0.984, respectively. We assured that the STRUCTURE analysis of 10 microsatellite loci with a *qi* of 0.95 was quite effective in distinguishing between admixed and pure individuals, whereas using criteria of a *qi* of 0.8, 0.95, or 0.99 proved ineffective in separating backcross hybrids from pure individuals, as demonstrated by samples PS57, IN74, and IN166. This exemplifies the limitations of the STRUCTURE program in identifying backcross hybrids^31^.

Based on the NEWHYBRIDS results, the majority of the hybrids that could be class-identified were F2 hybrids (~ 35%) and backcrosses with *M. leucocephala* (~ 9%), respectively, implying that F1 hybrids are more likely to mate with each other than with the parental species. In other words, morphologically intermediate F1 hybrids appear to be selected infrequently by pure parentals. Furthermore, some hybrids are more prone to backcrossing with *M. leucocephala* than *M. cinerea*. There are two possible explanations for this: (1) pure *M. cinerea* exhibit strong premating isolation and so do not choose to mate with morphologically intermediate F1 hybrids; (2) *M. leucocephala* outnumbers *M. cinerea* in DZ, increasing the likelihood of encountering and mating between the F1 hybrid and *M. leucocephala*. The latter point is in accord with a greater number of backcrosses with *M. cinerea* (nine individuals) than with *M. leucocephala* (one individual) being previously detected at Jurong Bird Park, where *M. cinerea* predominated over *M. leucocephala*^25^*.* As a result, this data supports the second explanation, where both *M. cinerea* and *M. leucocephala* exhibit weak mate recognition and discrimination.

We discovered cases of discrepancy between the morphological and genetic data, as demonstrated by sample PS57 that exhibited *M. leucocephala* phenotypic characteristics, but the genetic data represented *M. cinerea*, emphasizing the limitations of morphological data in distinguishing hybrids from pure species. This individual is probably a backcross with *M. cinerea*, which carries the genes responsible for the formation of *M. leucocephala* plumage traits. Since morphological traits may be regulated by several genes associated with the selection and fitness of the parental species^32^. Therefore, further genetic studies are required to consider which genes control the morphological characteristics of hybrids and the two stork species^33^. In addition to the above example, morphological-genetic incongruities were discovered in nine intermediate individuals (IN03, IN17, IN21, IN70, IN100, IN108, IN109, IN128, and IN135) that were genetically identified as *M. leucocephala*, but phenotypically they do not appear to be pure *M. leucocephala*. We suggest that analyses of a large number of genetic markers is required to resolve these ambiguous results^31,34–36^.

Overall, the morphological and genetic results were relatively consistent, and the proportion of hybrids that morphologically resembled *M. leucocephala* was greater than that of hybrids that resembled *M. cinerea*. Among the morphologically intermediate individuals detected in the DZ population, F2 hybrids and backcrosses with *M. leucocephala* showed similar morphological features that are intermediate between the pure parentals; all of them had a few very pale pinkish tertial feathers, five (56%) individuals had unclear black and white striped markings on the wings [the estimated MOW areas were less than the lowest value (366 cm^2^) detected in pure individuals], and none of them had a clear black bar across the breast; while three and one individuals of F2 hybrids and backcross with *M. leucocephala*, respectively, lacked this trait.

In contrast, hybrids of an unknown generation had a similar appearance to pure *M. leucocephala*, except that none of them had a clear black bar across the breast. As a result, the absence or appearance of an unclear black bar across the breast may be linked to a genetic admixture in hybrid genomes, and so this trait can be used to initially visually distinguish hybrids from pure individuals. Note that the diagnostic phenotypic traits used in the study are only applicable at the adult stage because juveniles of the two stork species (including hybrid individuals) exhibit a similar appearance of a brownish plumage and look different from adults^13,24^. To preserve the genomic integrity of the pure species, the multilocus markers obtained in this study could be utilized as diagnostic markers to detect hybrids of *M. cinerea* and *M. leucocephala* at any stage of age (nestlings, juveniles, or adults) in both captive and wild populations.

### Conservation and management implications

Human disturbance is regarded as one of the major factors causing hybridization and subsequent genomic contamination between closely related species, particularly in captivity, such as in zoos^4,37^. This study revealed that the endangered *M. cinerea* had genomic contamination from *M. leucocephala* and vice versa during its 16 years in captivity in Thailand through repeated backcrossing. Three (6.25%) of the 48 storks from NRZ and 38 (57.58%) of the 66 storks from DZ had hybrid identities. These hybrids are now kept in zoos and can be individually identified through mark recapture rings. To prevent crossbreeding, we recommend that hybrid storks be kept separate from pure storks and that they should not be used as stocks in future breeding or reintroduction programs. If hybrids must be raised in the same enclosure as their pure parental species (i.e., due to limited zoo space or insufficient birdcages for separation), sterilizing both the male and female hybrids is another option for preventing genetic contamination and preserving the genetic integrity of pure species. Moreover, the discovery of such backcrosses serves as a warning that the remaining individuals of *M. leucocephala* at DZ should urgently be genetically investigated to ensure each individual’s genomic purity.

In addition to the detection of hybrids in captivity, there have also been occasional reports of storks with intermediate trait detections in some wetland habitats in Thailand, such as at Bueng Boraphet (Nakhon Sawan Province), Bang Pu recreation center (Samut Prakan Province), Khlong Tamru salt pans (Chon Buri Province), Bang Tabun (Phetchaburi Province), and Laem Phak Bia (Phetchaburi Province)^38^. It is possible that these birds are natural hybrids, or they are captive hybrids that have been released or escaped from captivity. So far, no genetic evaluation is available to confirm that those individuals are hybrids of *M. cinerea* and *M. leucocephala* or not, and so it is imperative that a thorough and urgent genetic investigation of these birds be conducted. Besides, other studies have shown that both *M. cinerea* and *M. leucocephala* can hybridize with other related stork species in captivity, such as the lesser adjutant *Leptoptilos javanicus*^13,24^. Zookeepers should be aware of this and avoid rearing them with *L*. *javanicus* or other related species. More importantly, habitat loss is one of the major threats and causes of the decline or extinction of wild large waterbirds^16,39,40^. In situ preservation and protection of wetland habitats is also recommended, not only for the future sustainable conservation of *M. cinerea* and *M. leucocephala*, but also to reduce the chance of hybridization in inevitable overlapping areas.

## Conclusion

A total of 41 hybrids (*M. cinerea* × *M. leucocephala*) were identified, comprised of three individuals from NRZ (*n* = 48) and 38 individuals from DZ (*n* = 66). Most hybrids could be classified according to their morphology as follows: a few pinkish tertial feathers, unclear black and white markings on the wings, and an absence of/or unclear black breast band. The latter trait is plainly apparent in hybrids and so can be used to visually distinguish hybrids from pure species. Combining morphological identification with genetic assessment (both mtDNA and nDNA) provides a more efficient approach for detecting and distinguishing hybrids from the parental species, and these findings can serve as an important foundation for conservation efforts and long-term management of *M. cinerea* and *M. leucocephala* populations.

## Methods

### Sampling and DNA extraction

Blood samples of 173 *M. leucocephala* (including putative hybrids) and 48 *M. cinerea* were collected in 2018 during regular veterinary examinations at two captive breeding locations, Dusit Zoo (DZ), Bangkok, and Nakhon Ratchasima Zoo (NRZ), Nakhon Ratchasima Province, respectively. Before blood was collected, all birds were weighed, rung with a mark recapture ring, and photographed for morphological analysis. Approximately 300 μL of blood was taken from the subclavian vein under the wing by the zoological park veterinarian team using a 1-mL syringe. Blood samples were immediately stored in a 1.5-mL tube coated with EDTA for prevention of blood coagulation and later preserved at − 80 °C in the laboratory until DNA extraction. This study was performed in accordance with relevant guidelines and regulations, including the ARRIVE guidelines (http://www.ARRIVEguidelines.org) for the ethics of animal research. All experimental protocols were approved by the Institutional Animal Care and Use Committee (IACUC), Faculty of Science, Chulalongkorn University (Protocol Review No. 1823015).

Total genomic DNA was extracted from dried blood spots on filter paper using a FavorPrep™ Tissue Genomic DNA Extraction Mini Kit (Favorgen Biotech Corp.) following the manufacturer’s instruction.

### Morphological analysis

**Figure 4:**
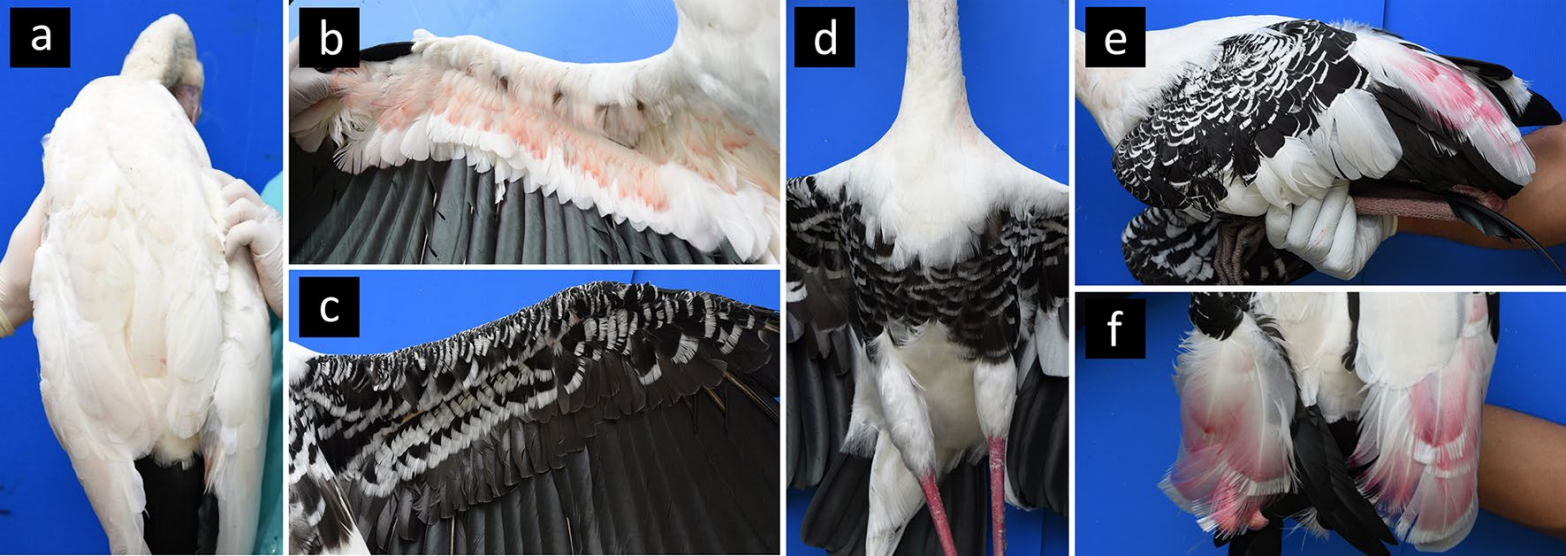
Six distinct phenotypic characteristics of (**a**,**b**) *M. cinerea* and (**c**–**f**) *M. leucocephala* for morphological analysis. (**a**) all-white body (AWB), (**b**) pink markings under the wings (PMUW), (**c**) black and white markings under the wings (MUW), (**d**) black bar across the breast (BBB), (**e**) black and white markings on the wings (MOW), and (**f**) pink tertial feathers (PTF).

Only 212 adult individuals (164 from DZ, 48 from NRZ) were morphologically analyzed because juveniles of the two species and their putative hybrids appear similar in their plumage patterns and colorations, making species discrimination implausible. To differentiate pure storks from the hybrids based on their morphological characters, we photographed each stork individually and compared their major differences on six distinguishable phenotypic traits, following with modification from Yee et al.^24^ and Nabhitabhata et al.^41^: 1; all-white body (AWB), 2; pink/coral markings under the wings (PMUW), 3; black and white markings under the wings (MUW), 4; black bar across the breast (BBB), 5; black and white markings on the wings (MOW), and 6; pink tertial feathers (PTF) (Fig. 4).

The AWB and PMUW traits were scored as 0 or 1 according to their absence or presence, respectively. For MUW, it was scored as 0, 1, or 2 for absent, systematic markings, and nonsystematic markings, respectively. For BBB, MOW, and PTF, these traits exhibit wide variations among individuals and could not be easily identified between morphologically pure *M. leucocephala* and putative hybrids. We, therefore, examined these three phenotypic traits in detail by calculating the marking/bar areas of those three traits from digital images using Adobe Photoshop CC 2018 and ImageJ v.1.51^42^, and then recording the area values instead of “absent” or “present” for each specimen. To ensure the same quality of the all-digital images, we standardized the scale bar of each image as 2 cm per one subdivision. The image was cropped to only cover the BBB. The width of each image was then determined to be equal to the width of the breast depending on each individual (approximately 50 cm), and the resolution of each image was set to 72 pixels/centimeters. The black area of the breast in a JPEG file was converted to the red area by adjusting the brightness using the threshold color tool in ImageJ. The resulting red area of the breast was then measured using the ROI Manager tool. Each BBB area was measured twice for each digital image and the “average” values used for further analyses. The same method was applied for measuring the black area of the MOW. For the PTF, the shades of pink were estimated using the Commission internationale de l’e´clairage L* a* b* (CIELab) color space technique in the Adobe Photoshop program. The CIELab color uses three values (L, a, and b) to specify color: the L-axis (lightness), the a-axis (green to red), and the b-axis (blue to yellow)^43,44^. We converted the RGB image to the Lab color image, then selected five locations covering the pink area of the feathers, and used the “average” of five positive a-axis values. In this case, the brightness and darkness of images were not standardized prior to estimation because these parameters have no effect on shades of pink values on the a-axis.

For data analysis, we used canonical discriminant function (CDF) analysis to predict the groups to which each individual was assigned based on the six phenotypic variables listed above, and to visualize the relationships between the group positions. The statistical analysis was performed using IBM SPSS Statistics for window^®^ version 26 (IBM Corporation).

### Mitochondrial DNA amplification and sequencing

A portion of the mitochondrial cytochrome* b* gene (*cyt b*) was PCR amplified and sequenced to identify the haplotype of each stork individual, as well as the maternity of the hybrids. This PCR amplification and sequencing used the L14990 and H16065 primers^45^ with thermal cycling performed in a 45 µL reaction volume containing 2 µL of DNA template (approximately 30–50 ng), 0.5 µM of each forward and reverse primer and 1 × premix of EmeraldAmp^®^ MAX PCR master mix (Takara). The PCR cycling conditions were as follows: initial denaturation at 93 °C for 3 min, followed by 35 cycles of denaturation at 93 °C for 30 s, annealing at 52 °C for 1 min, and extension at 72 °C for 1.15 min, and then followed by a final extension at 72 °C for 10 min. The PCR products were electrophoresed using a 0.8% (w/v) agarose gel, stained with SYBR^®^ Safe DNA gel staining dye (Invitrogen™), and visualized under a blue light transilluminator. The desired PCR products were sent to Bionics Inc., South Korea for commercial purification via gel extraction and sequencing services.

### Microsatellite genotyping

A total of 114 stork specimens, which were phenotypically classified as pure *M. leucocephala* (*n* = 24), intermediate individuals (*n* = 37), and pure *M. cinerea* (*n* = 53), were genotyped at 14 microsatellite loci that were originally developed from (i) the oriental white stork *Ciconia boyciana*: Cbo108, Cbo109, Cbo133, Cbo151, Cbo168, and Cbo235^46^, and (ii) the white stork *Ciconia ciconia*: Cc05, Cc06, Cc07, Cc10, Cc42, Cc50, Cc58, and Cc72^47,48^. The 5’- end of all forward primers was labeled with one of two fluorescent dyes (6-FAM or HEX, Macrogen Inc. and Bionics Inc., South Korea) to facilitate automated genotyping. Some markers were amplified in a multiplex, whereas others were amplified independently. PCR amplifications were performed in a reaction volume of 15 µL, containing 1 × premix of EmeraldAmp^®^ MAX PCR master mix (Takara), 0.5 µM of each forward and reverse primer and 30–50 ng of genomic DNA. Amplification profiles for all primers were conducted as follows: initial denaturation at 94 °C for 5 min, followed by 35 cycles of denaturation at 94 °C for 30 s, annealing for 30 s (see detail in Supplementary Table S5 for locus-specific annealing temperature), and extension at 72 °C for 40 s, and then followed by a final extension at 72 °C for 10 min. The amplicons were resolved on a 3% (w/v) agarose gel by electrophoresis. The desired fluorescent-labeled PCR products were then sent to Bionics Inc. in South Korea for genotyping using Rox-500 as an internal size standard in each capillary. Allele sizes were determined using GeneMaker^®^ software v.2.6.4 (SoftGenetics, LLC).

### Phylogenetic analyses on mtDNA data

All obtained nucleotide sequences were edited and aligned using CLUSTAL W^49^ implemented in MEGA v.11.0.6^50^, and the sequences were translated into amino acids to check for gaps or internal stop codons. We generated the phylogenetic trees by using both Maximum likelihood (ML) and Bayesian inference (BI) methods to analyze phylogenetic relationships and mtDNA haplotypes. The best fit model of nucleotide substitution for the data set of *cyt b* sequences was estimated using jModelTest v.2.1.10^51^ based on the Bayesian Information Criteria (BIC). jModelTest indicated the best model of nucleotide substitution was GTR + G. The ML tree was performed in PhyML v.3.0^52^, using the GTR + G model and 1,000 bootstrap replicates. The BI was conducted in MrBayes v.3.2.1^53^, performing 10,000,000 generations with sampling every 100 steps and a burn-in of 2,500 generations. The results were visualized in Figtree v.1.4.4^54^. The *cyt b* sequences of *M. ibis* (Genbank accession: U72784.1) and *M. americana* (AF082066.2), the two closely related species of our interest, were used as outgroups. We additionally included two retrieved *cyt b* sequences of *M. cinerea* (U72778.1) and *M. leucocephala* (U72777.1) from GenBank in the phylogenetic analyses.

### Population structure and hybridization analyses on microsatellite data

The microsatellite data was tested for departures from Hardy–Weinberg equilibrium (HWE) of loci and within the population using GENEPOP v.4.7^55^. The extent of linkage disequilibrium between pairs of loci was evaluated using FSTAT v.2.9.4^56^ with Bonferroni correction at a significance level of 0.01. To test for hybridization, we first used the Bayesian clustering procedure implemented in STRUCTURE v.2.3.3^31^ to identify genetically distinct clusters (K values) and potential admixture between species by calculating the posterior probability. In addition, STRUCTURE was used under the admixture model with correlated allele frequencies and without including sampling location as a prior. We used 10 replicates for each value of K, ranging from K = 1 to K = 10, with 2,000,000 MCMC replications after burn-in following a burn-in period of 500,000. The best value of K was selected following the Delta K (ΔK) proposed by Evanno et al.^57^ method, as calculated in STRUCTURE HARVESTER. A threshold membership coefficient (*qi*) value of 0.95 was used to distinguish between pure individuals (0 < *qi* < 0.05 or 0.95 < *qi* < 1) and hybrids (0.05 < *qi* < 0.95).

To considered if an individual had genomic admixture between two species (a hybrid), Bayesian posterior probability was applied using NEWHYBRIDS v.1.1^35^ to identify pure individuals and to classify hybrids into one of the six distinct frequency classes: pure *M. leucocephala* (PS), pure *M. cinerea* (MS), first generation hybrid (F1), second generation hybrid (F2), backcross of F1 with PS (BxPS) and backcross of F1 with MS (BxMS). The NEWHYBRIDS was run for five replicates with 1,000,000 sweeps and values after a burn-in period of 500,000 sweeps using Jeffreys-type prior for Theta and Pi. Individuals were assigned to NEWHYBRIDS genotype classes based on their posterior probability values (*P*), with *P* > 0.5 indicating that they belonged to that class. If no value reached 0.5 but the total of all hybrid classes was greater than this threshold, individuals were classified as hybrids of unknown generation.

## Supplementary Information

Supplementary Tables.

## Data Availability

The datasets generated and analyzed in the current study are available from the corresponding author on reasonable request. Mitochondrial *cyt b* sequences are available at the NCBI GenBank accession numbers OP985458-OP985461.
