# Supplementary material for: Detecting introgressive hybridization to maintain genetic integrity in endangered large waterbird: a case study in milky stork
Source: Sci Rep. 2023 Jun 1;13:8892. doi: 10.1038/s41598-023-35566-x (PMC10235083; doi:10.1038/s41598-023-35566-x)
Supplement: Supplementary file 1 — Supplementary Tables. [file 41598_2023_35566_MOESM1_ESM.pdf]

Detecting introgressive hybridization to maintain genetic integrity in endangered large waterbird: a case study in milky stork

Damisa Kaminsin[1], Natapot Warrit[2], Rangsinee Sankhom[3], Krittee Piamsanga[1], Saowaphang Sanannu[4], Sudarath Baicharoen[5], and Amporn Wiwegweaw[1,*]

[1] Population and Conservation Genetics Laboratory, Department of Biology, Faculty of Science, Chulalongkorn University, Bangkok 10330, Thailand

[2] Center of Excellence in Entomology and Department of Biology, Faculty of Science, Chulalongkorn University, Bangkok 10330, Thailand

[3] Durrell Institute of Conservation and Ecology, School of Anthropology and Conservation, University of Kent, Canterbury, Kent CT2 7NR, United Kingdom

[4] Genomic Resource Center, Animal Conservation and Research Institute, The Zoological Park Organization of Thailand, Bangkok 10300, Thailand

[5] Genetic Center, Animal Conservation and Research Institute, The Zoological Park Organization of Thailand, Bangkok 10300, Thailand

*Corresponding author: amporn.w@chula.ac.th

Supplementary Table S1. Eigenvalues and standardized canonical discriminant function coefficients used in the plot.

	**Function 1**	**Function 2**
**Eigenvalue**	11.938	1.684
**Cumulative %**	87.6	12.4
Black bar across the breast (BBB)	-0.465	0.794
Black and white markings on the wings (MOW)	-0.140	0.025
Pink tertial feathers (PTF)	-0.252	0.318
All-white body (AWB)	2.498	2.751
Black and white markings under the wings (MUW)	2.362	2.606
Pink markings under the wings (PMUW)	-0.360	-0.390

Supplementary Table S2. An overview of fragment analysis results of 14 microsatellite loci between *M. leucocephala* and *M. cinerea* populations.

**Locus**	**Number of alleles**	**Allele size (bp)**	
		M. leucocephala	*M. cinerea*
Cbo108	5	126-139	133-137
Cbo109	2	173-175	173-175
Cbo133	1	155	155
Cbo151	4	141-147	143-147
Cbo168	2	120-122	120
Cbo235	1	160	160
Cc05	1	154	154
Cc06*	-	-	-
Cc07	12	350-400	350-390
Cc10	6	90-114	90-114
Cc42	5	302-318	306-318
Cc50	2	194-200	194-200
Cc58	5	212-232	220-234
Cc72	3	195-199	195-199

* Cc06 was unreliable reading from GeneMaker® software v.2.6.4.

Supplementary Table S3. The value of delta K (ΔK) according to the Evanno method from K=1 to K=10.

K	Replications	Mean LnP(K)	Stdev LnP(K)	Delta K
1	10	-2119.03	0.62	-
2	10	-1931.36	1.22	63.09
3	10	-1820.45	0.46	210.75
4	10	-1806.50	10.02	0.10
5	10	-1791.55	4.03	27.96
6	10	-1889.16	19.22	0.78
7	10	-2001.88	57.08	2.57
8	10	-1967.94	39.32	1.23
9	10	-1982.52	40.55	0.30
10	10	-2009.08	31.70	-

Supplementary Table S4. Individuals identified as hybrids, pure *M. cinerea* in DZ, unidentified, pure *M. leucocephala*, and pure *M. cinerea* inferred from morphology, mtDNA and microsatellite data.

No.	Individual ID.	Location	Sex	mtDNA	STRUCTURE	NEWHYBRIDS
Hybrids						
1	PS06	DZ	♀	PS	Admixed (0.840)	Hybrid*
2	PS18	DZ	♀	PS	Admixed (0.925)	PS (0.517)
3	PS30	DZ	♀	PS	Admixed (0.923)	BxPS (0.517)
4	PS57	DZ	♂	MS	MS (0.025)	MS (0.984)
5	PS59	DZ	♀	PS	Admixed (0.10)	Hybrid*
6	PS61	DZ	♂	PS	Admixed (0.698)	Hybrid*
7	PS67	DZ	♂	PS	Admixed (0.493)	F2 (0.525)
8	PS75	DZ	♂	PS	Admixed (0.702)	F2 (0.588)
9	PS167	DZ	♀	MS	Admixed (0.902)	Hybrid*
10	IN04	DZ	♀	PS	Admixed (0.737)	Hybrid*
11	IN11	DZ	♂	PS	Admixed (0.938)	PS (0.570)
12	IN23	DZ	♀	PS	Admixed (0.907)	Hybrid*
13	IN24	DZ	♂	PS	Admixed (0.917)	Hybrid*
14	IN26	DZ	♀	PS	Admixed (0.943)	PS (0.535)
15	IN32	DZ	♀	PS	Admixed (0.662)	F2 (0.741)
16	IN40	DZ	♀	PS	Admixed (0.799)	Hybrid*
17	IN44	DZ	♀	MS	Admixed (0.876)	Hybrid*
18	IN58	DZ	♀	PS	Admixed (0.575)	F2 (0.545)
19	IN74	DZ	♀	MS	MS (0.024)	MS (0.961)
20	IN83	DZ	♀	PS	Admixed (0.929)	Hybrid*
21	IN87	DZ	♂	PS	Admixed (0.877)	Hybrid*
22	IN88	DZ	♀	PS	Admixed (0.930)	Hybrid*
23	IN98	DZ	♂	PS	Admixed (0.729)	F2 (0.608)
24	IN93	DZ	♀	MS	Admixed (0.147)	MS (0.741)
25	IN101	DZ	♂	PS	Admixed (0.819)	BxPS (0.584)
26	IN111	DZ	♂	MS	Admixed (0.933)	PS (0.516)
27	IN119	DZ	♀	PS	Admixed (0.785)	F2 (0506)
28	IN123	DZ	♀	MS	Admixed (0.759)	Hybrid*
29	IN124	DZ	♀	PS	Admixed (0.747)	F2 (0.887)
30	IN133	DZ	♂	MS	Admixed (0.87)	Hybrid*
31	IN134	DZ	♂	PS	Admixed (0.675)	PS (0.507)
32	IN142	DZ	♂	PS	Admixed (0.832)	F2 (0.529)
33	IN156	DZ	♀	PS	Admixed (0.496)	F2 (0.569)
34	IN163	DZ	♀	MS	Admixed (0.532)	F2 (0.557)
35	IN166	DZ	♂	MS	MS (0.050)	MS (0.951)
36	IN168	DZ	♂	MS	Admixed (0.939)	Hybrid*
37	IN169	DZ	♀	MS	Admixed (0.179)	MS (0.882)
38	MS172	DZ	♂	MS	Admixed (0.839)	BxPS (0.609)
39	MS21	NRZ	♂	MS	Admixed (0.797)	F2 (0.562)
40	MS26	NRZ	♀	MS	Admixed (0.944)	Hybrid*
41	MS47	NRZ	♀	MS	Admixed (0.934)	Hybrid*
Pure *M. cinerea* in DZ						
1	MS78	DZ	♀	MS	MS (0.024)	MS (0.972)
2	MS85	DZ	♀	MS	MS (0.014)	MS (0.966)
3	MS96	DZ	♀	MS	MS (0.018)	MS (0.926)
4	MS177	DZ	♂	MS	MS (0.008)	MS (0.996)

No.	Individual ID.	Location	Sex	mtDNA	STRUCTURE	NEWHYBRIDS
Unidentified individuals						
1	IN03	DZ	♂	PS	PS (0.973)	PS (0.835)
2	IN17	DZ	♂	PS	PS (0.954)	PS (0.632)
3	IN21	DZ	♀	PS	PS (0.960)	PS (0.616)
4	IN70	DZ	♂	PS	PS (0.955)	PS (0.671)
5	IN100	DZ	♂	PS	PS (0.974)	PS (0.852)
6	IN108	DZ	♀	PS	PS (0.980)	PS (0.854)
7	IN109	DZ	♀	PS	PS (0.979)	PS (0.848)
8	IN128	DZ	NA	PS	PS (0.977)	PS (0.674)
9	IN135	DZ	♀	PS	PS (0.954)	PS (0.608)
Pure *M. leucocephala*						
1	PS19	DZ	♀	PS	PS (0.925)	PS (0.859)
2	PS22	DZ	♀	PS	PS (0.960)	PS (0.660)
3	PS25	DZ	♂	PS	PS (0.973)	PS (0.799)
4	PS27	DZ	♀	PS	PS (0.977)	PS (0.832)
5	PS31	DZ	♂	PS	PS (0.961)	PS (0.698)
6	PS34	DZ	♀	PS	PS (0.970)	PS (0.798)
7	PS38	DZ	♂	PS	PS (0.976)	PS (0.834)
8	PS39	DZ	♀	PS	PS (0.979)	PS (0.829)
9	PS53	DZ	♂	PS	PS (0.965)	PS (0.708)
10	PS56	DZ	♂	PS	PS (0.974)	PS (0.780)
11	PS62	DZ	♀	PS	PS (0.982)	PS (0.845)
12	PS73	DZ	♀	PS	PS (0.983)	PS (0.910)
13	PS82	DZ	♀	PS	PS (0.976)	PS (0.828)
14	PS125	DZ	♂	PS	PS (0.971)	PS (0.811)
15	PS131	DZ	♂	PS	PS (0.964)	PS (0.817)
Pure *M. cinerea*						
1	MS1	NRZ	NA	MS	MS (0.982, cluster 1)	MS (0.996)
2	MS2	NRZ	NA	MS	MS (0.975, cluster 1)	MS (0.965)
3	MS3	NRZ	NA	MS	MS (0.976, cluster 1)	MS (0.993)
4	MS4	NRZ	NA	MS	MS (0.920, cluster 1)	MS (0.910)
5	MS5	NRZ	NA	MS	MS (0.920, cluster 1)	MS (0.910)
6	MS6	NRZ	NA	MS	MS (0.981, cluster 1)	MS (0.995)
7	MS7	NRZ	NA	MS	MS (0.851, cluster 1)	MS (0.988)
8	MS8	NRZ	NA	MS	MS (0.982, cluster 2)	MS(0.933)
9	MS9	NRZ	NA	MS	MS (0.975, cluster 2)	MS (0.975)
10	MS10	NRZ	NA	MS	MS (0.976, cluster 2)	MS (0.995)
11	MS11	NRZ	NA	MS	MS (0.840, cluster 1)	MS (0.932)
12	MS12	NRZ	NA	MS	MS (0.968, cluster 1)	MS (0.937)
13	MS13	NRZ	NA	MS	MS (0.981, cluster 2)	MS (0.992)
14	MS14	NRZ	NA	MS	MS (0.913, cluster 2)	MS (0.805)
15	MS15	NRZ	NA	MS	MS (0.973, cluster 2)	MS (0.997)
16	MS16	NRZ	NA	MS	MS (0.978, cluster 2)	MS (0.948)
17	MS17	NRZ	NA	MS	MS (0.746, cluster 1)	MS (0.978)
18	MS18	NRZ	♂	MS	MS (0.954, cluster 2)	MS (0.988)
19	MS19	NRZ	NA	MS	MS (0.977, cluster 1)	MS (0.971)
20	MS20	NRZ	♂	MS	MS (0.908, cluster 2)	MS (0.790)
21	MS22	NRZ	♂	MS	MS (0.984, cluster 1)	MS (0.987)
22	MS23	NRZ	NA	MS	MS (0.974, cluster 1)	MS (0.990)
23	MS24	NRZ	♀	MS	MS (0.979, cluster 1)	MS (0.984)
24	MS25	NRZ	NA	MS	MS (0.975, cluster 1)	MS (0.990)

No.	Individual ID.	Location	Sex	mtDNA	STRUCTURE	NEWHYBRIDS
25	MS27	NRZ	NA	MS	MS (0.975, cluster 2)	MS (0.987)
26	MS28	NRZ	♀	MS	MS (0.978, cluster 1)	MS (0.977)
27	MS29	NRZ	♂	MS	MS (0.957, cluster 2)	MS (0.926)
28	MS30	NRZ	NA	MS	MS (0.955, cluster 2)	MS (0.994)
29	MS31	NRZ	♂	MS	MS (0.964, cluster 2)	MS (0.990)
30	MS32	NRZ	♂	MS	MS (0.975, cluster 2)	MS (0.997)
31	MS33	NRZ	NA	MS	MS (0.972 (cluster 2)	MS (0.992)
32	MS34	NRZ	NA	MS	MS (0.974, cluster 1)	MS (0.970)
33	MS35	NRZ	♀	MS	MS (0.935, cluster 1)	MS (0.950)
34	MS36	NRZ	NA	MS	MS (0.960, cluster 1)	MS (0.846)
35	MS37	NRZ	NA	MS	MS (0.975, cluster 1)	MS (0.987)
36	MS38	NRZ	NA	MS	MS (0.975, cluster 1)	MS (0.956)
37	MS39	NRZ	♀	MS	MS (0.968, cluster 2)	MS (0.856)
38	MS40	NRZ	♀	MS	MS (0.882, cluster 1)	MS (0.961)
39	MS41	NRZ	♀	MS	MS (0.972, cluster 2)	MS (0.990)
40	MS42	NRZ	♀	MS	MS (0.930, cluster 2)	MS (0.982)
41	MS43	NRZ	NA	MS	MS (0.976, cluster 1)	MS (0.954)
42	MS44	NRZ	♂	MS	MS (0.907, cluster 1)	MS (0.938)
43	MS45	NRZ	♀	MS	MS (0.972, cluster 1)	MS (0.964)
44	MS46	NRZ	♂	MS	MS (0.919, cluster 2)	MS (0.952)
45	MS48	NRZ	♂	MS	MS (0.954, cluster 2)	MS (0.717)

The individual's ID column labeled ‘PS’, ‘IN’, and ‘MS’ represent morphologically identified as *M. leucocephala*, intermediate individuals, and *M. cinerea*, respectively. DZ and NRZ represent Dusit Zoo and Nakhon Ratchasima Zoo, respectively. The mtDNA column labeled ‘PS’ and ‘MS’ represent haplotype inferred to originate from *M. leucocephala* and *M. cinerea*, respectively. The values in the column of STRUCTURE and NEWHYBRIDS are the membership coefficient (qi) and the posterior probability (P) of each individual analyzed from microsatellite data. Hybrid* is a hybrid of unknown generation. NA is no data available.

Supplementary Table S5. Nucleotide sequences and annealing temperatures (T_a) of 14 microsatellite primers used in this study.

Locus	Nucleotide sequence (5'→ 3')	T_a (°C)	Reference
Cbo108[a]	F:6-FAM-CCCAGGTCACAAATTATACG<br>R:GAGCCTCACAAAGTTCCCTG	55	Wang et al., 2011
Cbo109[a]	F: 6-FAM-GTGGTGTAGTCCAGTTTATG<br>R:ATAACACATGAATGACCTGG	55	Wang et al., 2011
Cbo133[a]	F: 6-FAM-GGACAAAAGGCGATTCTAGC<br>R:TTGAGCCAAACATCCGACAC	55	Wang et al., 2011
Cbo151[b]	F: 6-FAM-AATCTGGTCTTGGTCCTTTC<br>R:GGTTTTACCCTCTGACACTG	55	Wang et al., 2011
Cbo168[b]	F: 6-FAM-GGGTGCAGTTGAATTAGAC<br>R:AATATTTTGGTTTGGTAAAC	55	Wang et al., 2011
Cbo235[b]	F: 6-FAM-TGGCTAAACATCTCCAAAC<br>R:TACAAGTAACGCAAGGGTAC	55	Wang et al., 2011
Cc05	F: 6-FAM-GGAGGAATTTCAGCAATGGA<br>R: TGGGAAACCAGGAAACTGTC	54	Shephard et al., 2009
Cc06	F: HEX-CTCGCTGTCTCCTCTGCTCT<br>R: GAACAGCAATATCGCATCTACA	61	Shephard et al., 2009
Cc07	F: 6-FAMGCATGAAAATGCATAGAGCAGA<br>R: CCACCGTTATGATCCTTTGG	55	Shephard et al., 2009
Cc10[c]	F: HEX-TGTGACAGATGCAAAGCTCC<br>R: GTGTTTACTAGTTGGCTGTTCC	66	Shephard et al., 2009
Cc42	F: 6-FAM-GCAGGAAAGGAGGAAAGGTG<br>R: GCATCACAGTATGCAAACGC	68	Feldman Turjeman et al., 2016
Cc50	F: HEX-CTAATCTGTCCTGCCCTCCC<br>R: CACAGAGCCAGCAAGACAAG	70	Feldman Turjeman et al., 2016
Cc58[c]	F: 6-FAM-ACGAGGGTTGCTTAAGGAGG<br>R: AAATCTGTGCGCCAACTCAC	66	Feldman Turjeman et al., 2016
Cc72	F: 6-FAM-CATTGAAGATACTGGGCAGCC<br>R: GATCCCTTCATCACCAGCAG	68	Feldman Turjeman et al., 2016

F; forward primer, R; reverse primer. The primer designed by identical letter were amplified in a multiplex.
